# Winners and Losers of Atlantification: The Degree of Ocean Warming Affects the Structure of Arctic Microbial Communities

**DOI:** 10.3390/genes14030623

**Published:** 2023-03-01

**Authors:** Antonia Ahme, Anabel Von Jackowski, Rebecca A. McPherson, Klara K. E. Wolf, Mario Hoppmann, Stefan Neuhaus, Uwe John

**Affiliations:** 1 Alfred Wegener Institute, Helmholtz Centre for Polar and Marine Research, 27570 Bremerhaven, Germany; 2 UMR7621 Laboratoire d’Océanographie Microbienne, CNRS/Sorbonne Université, 66650 Banyuls-sur-Mer, France; 3 Institute for Marine Ecosystem and Fisheries Science, University of Hamburg, 22767 Hamburg, Germany

**Keywords:** Fram Strait, West Spitsbergen Current, incubation experiment, species composition, traits, thermal limits, cell size, trophic mode, pelagic microorganisms, microplankton

## Abstract

Arctic microbial communities (i.e., protists and bacteria) are increasingly subjected to an intrusion of new species via Atlantification and an uncertain degree of ocean warming. As species differ in adaptive traits, these oceanic conditions may lead to compositional changes with functional implications for the ecosystem. In June 2021, we incubated water from the western Fram Strait at three temperatures (2 °C, 6 °C, and 9 °C), mimicking the current and potential future properties of the Arctic Ocean. Our results show that increasing the temperature to 6 °C only minorly affects the community, while an increase to 9 °C significantly lowers the diversity and shifts the composition. A higher relative abundance of large hetero- and mixotrophic protists was observed at 2 °C and 6 °C compared to a higher abundance of intermediate-sized temperate diatoms at 9 °C. The compositional differences at 9 °C led to a higher chlorophyll a:POC ratio, but the C:N ratio remained similar. Our results contradict the common assumption that smaller organisms and heterotrophs are favored under warming and strongly indicate a thermal limit between 6 °C and 9 °C for many Arctic species. Consequently, the magnitude of temperature increase is a crucial factor for microbial community reorganization and the ensuing ecological consequences in the future Arctic Ocean.

## 1. Introduction

The Arctic ecosystem is dramatically changing and increasingly influenced by the Atlantic Ocean due to a weakening of the Atlantic Meridional Overturning Circulation [1]. This so-called “Atlantification” implies a northward expansion of Atlantic water into the Arctic Basin, resulting in an increase in temperature and salinity, rapid sea ice decline, as well as an intrusion of temperate species [2]. In particular, the West Spitsbergen Current is the largest driver of Atlantification by transporting Atlantic water northwards [2]. Over the last decades, the Atlantic water in the Fram Strait and the West Spitsbergen Current has been steadily warming [3]. Approximately half of Atlantic water transport carried northwards by the West Spitsbergen Current recirculates in the Fram Strait [4] and eventually becomes part of the southward outflow of polar water, namely the East Greenland Current [5]. These dynamic and mixed properties make the Fram Strait and West Spitsbergen Current an ideal place to study water that is representative of an Arctic Ocean increasingly exhibiting Atlantic characteristics.

In addition to Atlantification, the Arctic is generally warming faster than the global average—a phenomenon referred to as Arctic amplification [6,7]. The co-occurrence of Atlantification and Arctic amplification is expected to affect the microbial community composition, as advected individuals may cope better with the new conditions than local species [8]. Anticipating shifts in community structure under abiotic change is complex and depends on various factors. Recently, trait differences among competing species have been identified as a good predictor for planktonic reorganization [9]. With regard to warming, traits such as the cell size [10] and the trophic mode [11] are known to affect the fitness and performance of a species, as they influence the thermal reaction norm for maximum growth [12].

Smaller cells were long believed to have an advantage under increasing temperatures as their supposedly higher mass-specific growth rates should enable them to outcompete larger cells [13]. Therefore, warming was expected to result in a community shift towards smaller species [14,15]. This assumption has been challenged by the repeated observation of growth rates peaking at intermediate cell sizes, even under higher temperatures [10,16,17]. However, in accordance with both theories, it is generally expected that comparably larger species suffer a competitive disadvantage when temperatures increase.

Another group that is assumed to experience a competitive disadvantage under warming is photoautotrophs. While the metabolism of phytoplankton is limited by their photosynthetic rate, heterotrophic plankton depends on food uptake and the rate at which it respires it [11]. Although all metabolic processes increase with temperature up to a point, the rate of increase is slower for photosynthesis compared to respiration. This is due to different temperature dependencies of the central chemical reactions—the production of ATP from glycolysis and the tricarboxylic acid cycle for respiration being more sensitive than Rubisco carboxylation for photosynthesis [18,19]. Therefore, several authors have suggested that while warming may also enhance phytoplankton growth, it disproportionally favors heterotrophic organisms [20,21,22]. Consequently, grazing pressure may increase [11,23].

Liu et al. [24] also found micro-grazers to be disproportionally advantaged by high temperatures but identified thermal optima as potential vectors for community response. This is in line with another set of studies that suggests most shifts in planktonic composition can be explained by the thermal niche of the respective species [12,25,26]. As thermal reaction norms are usually the result of adaptation and often reflect the biogeography of species [27,28,29], temperate organisms should have higher optimum and maximum temperatures for growth than polar organisms. This assumption is confirmed by the ongoing expansion of temperate species into the Arctic realm [30]. However, whether temperate or polar species will prevail may depend on their thermal optima and limits relative to the actual temperature increase occurring in the Arctic.

Currently, the degree of warming in Arctic waters remains unclear [6] but may be crucial in determining planktonic reorganization. This is particularly important considering the presence of temperate species with other metabolic traits, which are advected via Atlantification. Still, studies on the consequences of concurrent warming and Atlantification of the Arctic Ocean for local plankton communities are scarce. The aim of this study was to experimentally simulate the effect of different temperature scenarios on the composition and characteristics of microbial communities (i.e., protists and bacteria) from Atlantic water inflow to the Arctic Ocean. We hypothesized that small temperate heterotrophs increase in relative abundance with rising temperatures. Furthermore, we expected the diversity to decrease with increasing temperatures due to the dominance of a few well-adapted species.

## 2. Materials and Methods

### 2.1. Study Site and Seawater Physical Properties

**Figure 1:**
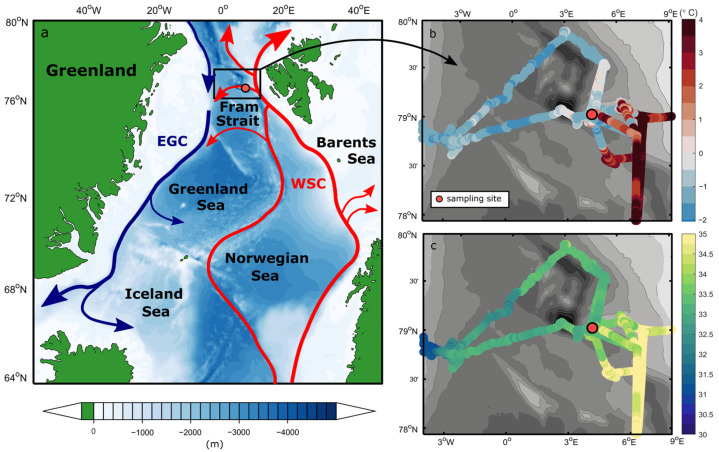
(**a**) Schematic of the Atlantic water circulation in the Nordic Seas and the near-surface (**b**) temperature and (**c**) salinity in the Fram Strait during PS126. The location of the sampling site HG-IV is highlighted as the red circle (4° 22.23′ W, 79° 4.86′ N). On the map, the red arrows represent the northwards flow of warm Atlantic water into the Arctic Ocean as the West Spitsbergen Current, with a large fraction of this water recirculating in the Fram Strait. The blue arrows denote the flow of modified Atlantic water southward within the East Greenland Current along the continental shelf break. The sketched currents are adapted from Beszczynska-Möller et al. [3].

Our experiment was performed on board the German research icebreaker RV Polarstern during the PS126 expedition to the long-term ecological research observatory Hausgarten in the Fram Strait in May/June 2021 [31]. To capture a plankton community with both Atlantic and Arctic characteristics, we chose a sampling site in the central Fram Strait (Figure 1a), where warm and salty Atlantic water recirculates and subducts under colder and fresher Polar water. The hydrographic properties of the water at this depth suggest that it is predominately mixed polar surface water (Figure S1), which is cold (<1 °C) and relatively fresh (34), overlaying an Atlantic water layer. However, the sample location shows high near-surface variability where warm (>0 °C) Atlantic waters often dominate (Figure 1b,c). Thus, it is anticipated that at least some of the species at the sample site are those found in Atlantic water and represent a background Atlantic community. A total of 30 L of seawater was collected from the chlorophyll maximum at 15 m at station HG-IV on 1 June 2021 (12:00 UTC, Figure S1). We used a ship-based SBE911 + CTD/rosette system (Sea-Bird Scientific, Washington, DC, USA) equipped with a standard suite of oceanographic sensors and 24 × 12 L OTE water sampling bottles. The sea-surface temperature and salinity were measured by two SBE21 thermosalinographs and two auxiliary SBE38 temperature sensors (Sea-Bird Scientific, Washington, DC, USA) installed in an underway seawater flow-through system on board the *Polarstern* [32]. The seawater inlet is located 11 m under the water’s surface. If not otherwise noted, salinity is always expressed after PSS-78 and is, therefore, unitless.

### 2.2. Experimental Set-Up

To remove metazoan plankton and ensure the same community composition within all treatments, we filtered the water through an ethanol-cleaned 150 µm mesh into an acid-cleaned 25 L carboy and gently mixed it. Water from the carboy was distributed evenly into nine autoclaved 2.3 L glass bottles (three temperature treatments in triplicates), which were closed with gas-tight lids. The remaining water was used to sample parameters of the starting community (t0) in triplicate. We took care not to introduce any bubbles during the filling procedure to prevent more fragile organisms, such as ciliates, from dying [33].

In order to keep cells in suspension, bottles containing the natural communities were mounted in triplicates onto three plankton wheels that turned at a speed of one round per minute. The plankton wheels stood in temperature-controlled incubation containers, which were kept at three different temperatures. We chose 2 °C as the lowest level because it was close to the mean temperature of 0.84 °C for the upper 100 m (Figure S1), and similar mean temperatures have also been recorded at this station previously [34]. The other two temperature levels of 6 °C and 9 °C were chosen to represent different scenarios of Arctic amplification (+4 °C and +7 °C respectively, [6]). All communities were exposed to 24 h artificial daylight with an irradiance of 30 µmol photons m^−2^ s^−1^ (SunStrip 35W fresh, ECONLUX GmbH, Köln, Germany) for ten days.

The nutrient concentrations in the field were low (0.06 ± 1.23 µM N$O_{3}^{-}$, 0.21 ± 0.08 µM P$O_{4}^{3-}$ , 0.08 ± 0.39 µM Si(OH)_4_); therefore, we added macro- and micronutrients to enable an investigation of the otherwise growth-limited photoautotrophic summer community. Nutrient pulses can also naturally occur through mixing events of short-term frontal systems and commonly enhance production in the surface waters of the eastern Fram Strait during summer [35,36]. Based on recommendations by Calbet and Saiz [37], we added 50 µM N$O_{3}^{-}$, 4.7 µM P$O_{4}^{3-}$, and 25 µM Si(OH)_4_, as well as trace metals and vitamins in accordance with the F/2 R medium concentrations. During the first days of the experiment, six bottles per temperature were incubated for a micrograzing experiment (not included in this dataset), with half of them undiluted and the other half diluted 1:5 with 0.22 µm filtered seawater taken from near the sampling location. After three days, we pooled the diluted and undiluted communities from t0 at each temperature. These pooled communities were then once again diluted (1:5), and nutrients were added as at t0 before they were incubated in triplicates for the last seven days of the experiment. Importantly, this did not result in any differences among our treatments, as community composition stayed stable at all temperatures during the first three days (Figure S2).

pH was measured at tfin using a pH meter (EcoScan pH 5, ThermoFisher Scientific, Waltham, MA, USA) with a glass electrode (Sentix 62, Mettler Toledo, Columbus, OH, USA) one-point calibrated with a technical buffer solution (pH 7, Mettler Toledo, Columbus, OH, USA). At t0 and tfin, samples for total alkalinity (TA) and dissolved nutrients were filtered through a 0.22 µm cellulose-acetate syringe filter (Nalgene, Rochester, NY, USA) and stored at 4 °C in 125 mL borosilicate bottles and 15 mL polycarbonate tubes, respectively. TA was measured by duplicate potentiometric titration using a TitroLine alphaplus autosampler (Schott Instruments, Mainz, Germany) and corrected with certified reference materials from A. Dickson (Scripps Institution of Oceanography, San Diego, CA, USA). The full carbonate system was calculated for tfin using the software CO2sys [38] with dissociation constants of carbonic acid by Mehrbach et al. [39], refitted by Dickson and Millero [40]. Dissolved nutrients were measured colorimetrically at t0 and tfin on a continuous-flow autoanalyzer (Evolution III, Alliance Instruments, Freilassing, Germany) following standard seawater analytical methods for nitrate and nitrite [41], phosphate [42], silicate [43], and ammonium [44].

### 2.3. Biomass Parameters

Biomass parameters were sampled in triplicate from t0 and from each replicate bottle at tfin. After thoroughly inverting the bottles, we vacuum-filtered (<−200 mbar) 300 mL for chlorophyll *a* (chl*a*), 200 mL for particulate organic carbon and nitrogen (POC/PON), and the same volume of sterile water for blanks onto pre-combusted glass-fiber filters (GF/F Whatman, Maidstone, UK). These were put into 2 mL cryovials (Sarstedt, Nümbrecht, Germany) and kept at −80 °C until processing. Filters for chl*a* were manually shredded in 6 mL of 90% acetone and extracted for 20 h at 8 °C according to the EPA method 445.0 [45]. The extract was centrifuged to remove residual filter snips, and chl*a* was determined on a Trilogy fluorometer (Turner Designs, San Jose, CA, USA) after correcting for phaeopigments via acidification (1 M HCl). Filters for POC/PON were also acidified (0.5 M HCl) and dried for 12 h at 60 °C. Analysis was performed using a gas chromatograph CHNS-O elemental analyzer (EURO EA 3000, HEKAtech, Wegberg, Germany). The chl*a*:POC ratio was calculated by dividing the chl*a* concentration by the POC concentration, and the C:N ratio was calculated by dividing the molar mass of POC by PON.

### 2.4. Community Composition and Diversity Analyses

Eukaryotic and bacterial community compositions were assessed by means of metabarcoding. A total of 500 mL of sample water was carefully vacuum-filtrated onto polycarbonate filters (0.8 µm nominal pore size, Nucleopore, Whatman, Maidstone, UK). Sequential filtration onto 0.2 µm pore size filters was not possible, which undoubtedly biased the analysis toward particle-associated heterotrophic bacteria. Filters were put into 2 mL cryovials containing 650 µL of warm extraction buffer and stored at −80 °C. After cell disruption with a MagNa Lyser (Roche Diagnostics, Basel, Switzerland), DNA extraction was performed according to the manufacturer’s protocol using the NucleoSpin Soil extraction kit (Macherey-Nagel GmbH, Düren, Germany). DNA concentration was quantified using a NanoDrop 8000 spectrophotometer (ThermoFisher Scientific, Waltham, MA, USA) and normalized to 5 ng µL^−1^. Amplicons of the variable region 4 (V4) of the 18S rRNA and 16S rRNA gene for eukaryotes and bacteria, respectively, were generated according to the standard protocol of amplicon library preparation (16S Metagenomic Sequencing Library Preparation, Part #15044223 Rev. B. Illumina, San Diego, CA, USA) using the forward primer CCAGCASCYGCGGTAATTCC and reverse primer ACTTTCGTTCTTGAT for 18S rRNA gene sequencing [46] and the forward primer GTGCCAGCMGCCGCGGTAA and reverse primer GGACTACHVGGGTWTCTAAT for 16S rRNA gene sequencing [47], all including an Illumina tail. Single samples were indexed using the Nextera XT Index Kit v2 Set A primers (Illumina, San Diego, CA, USA), and the resulting libraries were pooled, one pool each for 18S and 16S rRNA gene sequencing. Sequencing was performed on a MiSeq sequencer (Illumina, San Diego, CA, USA), producing 300 base pair paired-end gene amplicon reads. Demultiplexing and FASTQ sequence file generation were carried out using the Generate FASTQ workflow of the MiSeq sequencer software. Primers were removed with v2.8 cutadapt [48], and further processing of the sequence data was performed using the v1.18 DADA2 R package [49]. In consideration of the read quality, which usually drops towards the 3′-end, the forward reads were trimmed after 240 to 260 base pairs, and the reverse reads were trimmed after 200 to 210 base pairs. For each pool, error rates were learned independently, and sequences were denoised. Paired-end reads were merged with a minimum overlap of 50 base pairs allowing 0 mismatches, and chimeras were predicted and removed. Taxonomic assignment of the resulting amplicon sequence variants (ASVs) was performed using the reference databases PR2 (v4.12.0) for eukaryotes and SILVA (v138) for bacteria [50,51].

For downstream analyses, ASVs with a count of less than ten reads in replicate sample means were removed, as well as potential contaminations, metazoans, and fungi (Table S1). After rarefaction to confirm a sufficient sequencing depth, all samples were scaled to the lowest depth as described by Beule and Karlovsky [52]. Lastly, ASVs were normalized by centered log ratio (CLR) transformation [53] after removing the zeros with multiplicative simple replacement [54]. Processing of the data was performed using R v4.21 [55] with RStudio v2022.07.2 [56] and the packages dplyr (v1.0.10), vegan (v2.6-2), SRS (v0.2.3), zCompositions (v1.4.0.1), propr (v4.2.6), easyCODA (v0.34.3), and BiodiversityR (v2.14.4).

Annotated species were grouped according to three different categories: cell size, trophic mode, and thermal niche (Tables S2 and S3). Cell size was differentiated into picoplankton (here defined as <2 µm), nanoplankton (here defined as 2–20 µm), and microplankton (>20 µm), and trophic mode was differentiated into heterotrophs, autotrophs, mixotrophs, and parasitic. The data of both categories were assembled through an unstructured literature search via WoRMS [57] based on previous categorizations by Hörstmann et al. [58] and Schneider et al. [59]. The classification of thermal niches into Arctic, Arctic-temperate and cosmopolitan was performed as described by Šupraha et al. [60]. Whenever categories were unclear, species were marked as uncategorized.

**Figure 2:**
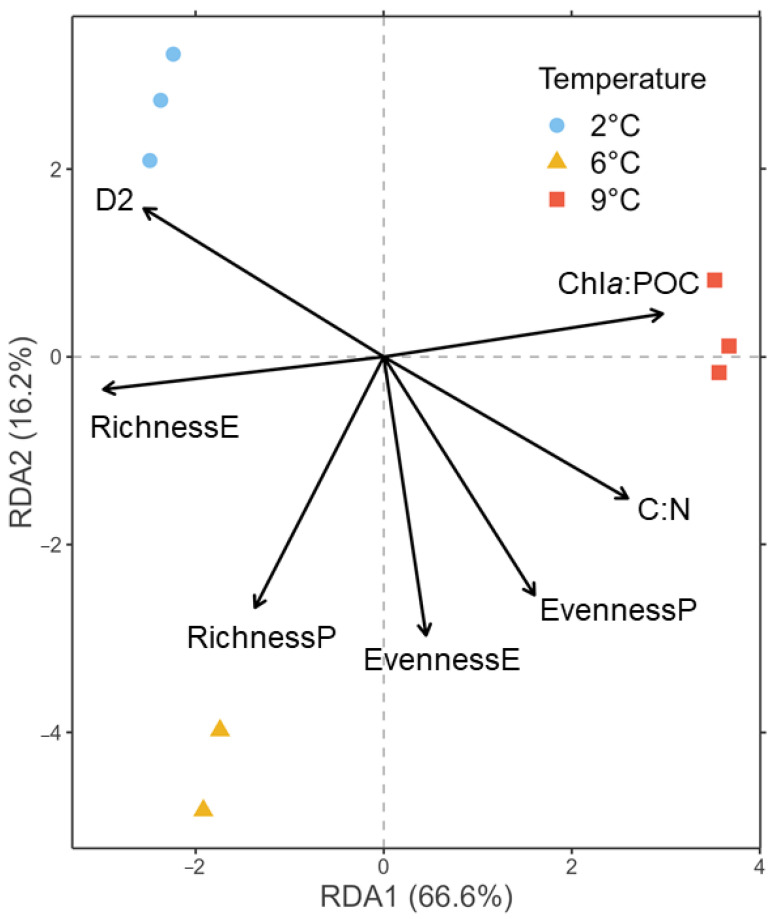
RDA of the CLR-transformed ASV counts of the 18S rRNA gene library color-coded to temperatures at tfin using biomass and diversity parameters as constraints represented by arrows. P = prokaryotes, E = eukaryotes, D2 = phenotypic diversity.

To obtain a measure for phenotypic diversity [61,62,63], 3.5 mL of the sample were preserved with hexamine-buffered formalin (0.5% final concentration) and stored at −80 °C after dark incubation for 15 min. For analysis, samples were thawed at room temperature, vortexed, and measured at a fast speed for three minutes using an Accuri C6 flow cytometer (BD Sciences, Franklin Lakes, NJ, USA) after setting the threshold of the FL-3 channel to 900. Phenotypic diversity (D2) was calculated for each sample based on the flow cytometric fingerprint according to Props et al. [61], using the values of FSC-H, SSC-H, FL-2, FL-3, and FL-4. To generate Figure 2, replicate C of the 6 °C treatment had to be removed due to an erroneous measurement of the flow cytometer and, thus, a missing D2 value. However, excluding D2 as a constraint, 6 °C replicate C clustered together with the other replicates, and the overall pattern did not change (Figure S4). Species richness and evenness were calculated from the sequencing data before transformation (Table S3).

### 2.5. Data Handling & Analyses

To examine the relative community composition, we took the replicate mean of the read abundance after normalization. ASV data grouped according to cell size, trophic mode, and thermal niche were analyzed after normalization and boxcox transformation by means of correspondence analysis (CA), according to Greenacre [64]. Furthermore, the parameters chl*a*:POC, C:N, D2, prokaryotic/eukaryotic richness, and evenness were used as explanatory matrices for a redundancy analysis (RDA) with the CLR-transformed ASV tables as response matrices at tfin. Before conducting any statistical tests, all parameters were checked for homogeneity of variance with Levene’s test and met the assumption. As the data of at least one temperature per parameter were not normally distributed, we log-transformed them and performed pairwise t-tests with Bonferroni correction to detect differences in biomass and diversity parameters between temperatures (Table S4). The choice of this test was based on a priori assumptions on existing differences between the temperatures based on our hypothesis. We checked whether the results would differ using one-way ANOVAs and post hoc Tukey’s tests (R Script on GitHub), and they did not. The significance level of all statistical tests was set to 0.05, and all data in the tables and text are shown as mean ± one standard deviation of the three replicates.

## 3. Results

### 3.1. Physical Ocean Properties and Water Masses

In the CTD profile taken at the time of sampling (Figure S1), a 20 m deep cold and fresh polar surface water layer overlaid a sharp pycnocline, with a warm Atlantic water layer at ~70 to 450 m. Near-surface temperature and salinity measured by the thermosalinograph throughout the experiment suggest strong variability in this part of the Fram Strait. During repeated visits to the sampling site, temperatures and salinities in the upper water column ranged between −1.6 °C and 4 °C and 32.5 and 35, respectively, in a period of only a few weeks (Figure 1b,c). The sampling site was located close to the Svalbard continental shelf, in a transition zone characterized by an Atlantic water recirculation regime (indicated in Figure 1a). There, conditions alternate between the warm Atlantic-influenced West Spitsbergen Current and the colder and fresher Polar water towards the central and western Fram Strait at a relatively high rate (see also von Appen et al. [65]). These findings suggest the presence of a highly variable and dynamic mixed polar surface water layer in the upper ~70 m (Figure S1), exhibiting properties of both Atlantic and Arctic waters over time.

### 3.2. Biomass and Diversity Parameters

During the ten days of incubation, the communities were neither nutrient- nor carbon-limited (Table S5). Chl*a*:POC was significantly higher and eukaryotic species richness was significantly lower at 9 °C than at 2 °C and 6 °C (Tables S3 and S4). The phenotypic diversity (D2) was significantly lower at 9 °C only compared to 2 °C. All other pairwise t-tests were not significant. The directions of change in all parameters in relation to temperature treatments are visualized in the RDA plot (Figure 2). Here, 66.6% of the variation is constrained by the first RDA axis, with 9 °C associated with a high chl*a*:POC ratio, while eukaryotic species richness increases in the direction of 2 °C and 6 °C. The two lower temperatures spread across the second RDA axis (16.2%), of which 2 °C was associated with high D2. Considering the t-tests, the effects of the C:N ratio, eukaryotic species evenness, and prokaryotic species richness can be considered negligible for the interpretation of the RDA (Table S4).

### 3.3. Community Composition—Eukaryotes

**Figure 3:**
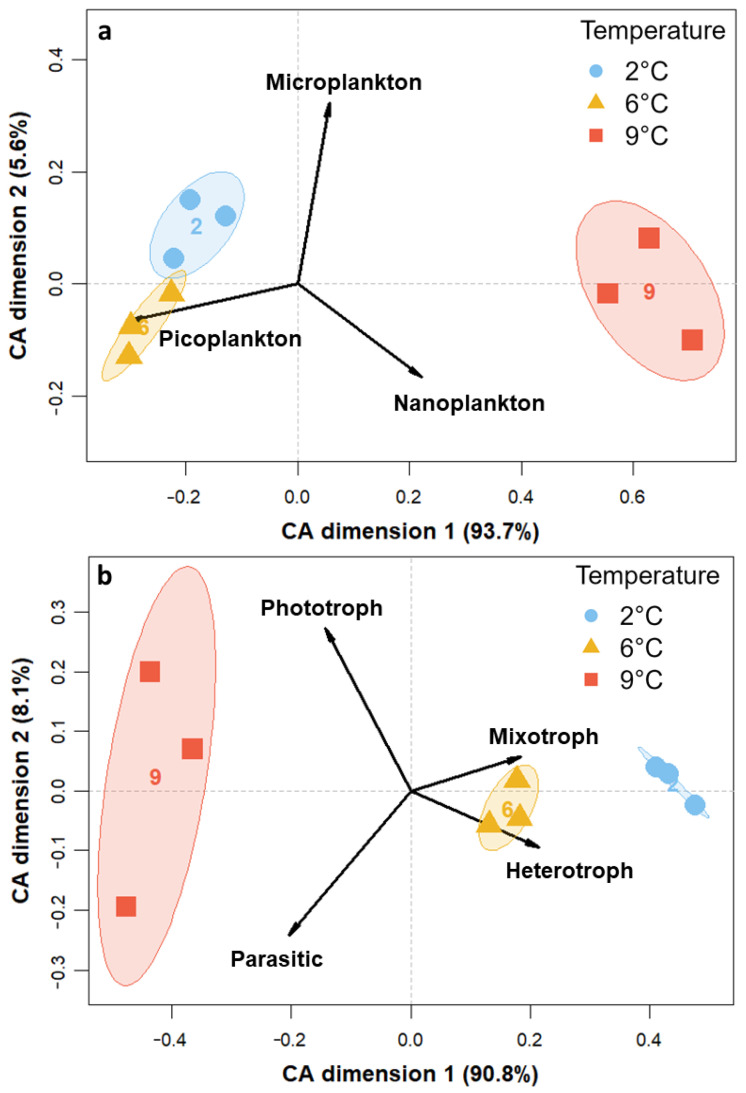

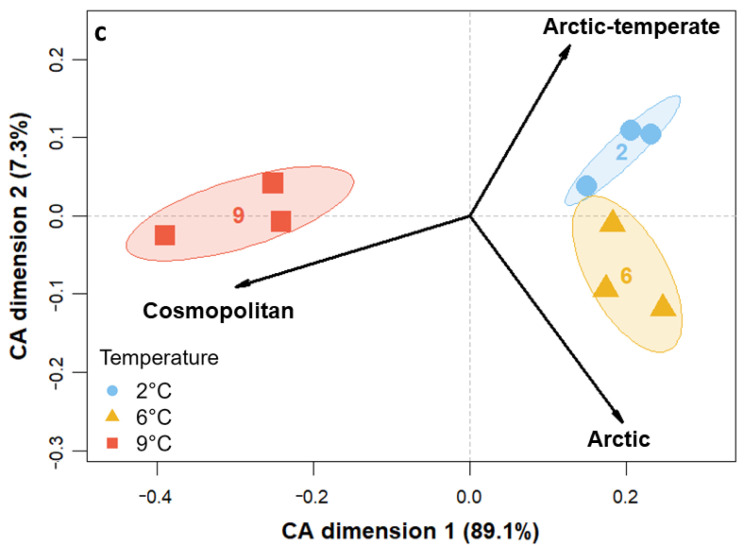
CA for the transformed read counts of the 18S rRNA gene library color-coded to temperatures at tfin constrained by (**a**) size classes, (**b**) trophic mode, and (**c**) thermal niche.

The size classes as well as trophic and thermal groups contributed to the variance among temperature treatments, as is evident from the CA plots in Figure 3. Generally, all replicates of the temperature treatments clustered together. Regarding size differences (Figure 3a), nanoplanktonic individuals (2–20 µm) mainly led to the clustering of 9 °C away from the two other temperatures on CA dimension one, with 93.7% of the variance constrained. On the second CA dimension (5.6%), 2 °C and 6 °C clustered away from each other based on differences in pico- and microplankton read abundances. In terms of trophic mode (Figure 3b), the three temperature treatments mainly spread out along the first axis (90.8%), with 6 °C and 2 °C on one side being gradually dominated more by hetero- and mixotrophy and 9 °C on the other side comprising more phototrophic and parasitic organisms. The replicates at 9 °C also spread out on the second axis (8.1%), along with the explanatory variables phototrophy and parasitism pointing in opposite directions. In the thermal niche CA (Figure 3c), 9 °C samples clustered away from 6 °C and 2 °C along the explanatory variable cosmopolitan on the first axis, which constrained 89.1% of the variance. The variables Arctic and Arctic-temperate pointed towards samples of the two colder temperatures and spread out slightly along the second axis (7.2%).

**Figure 4:**
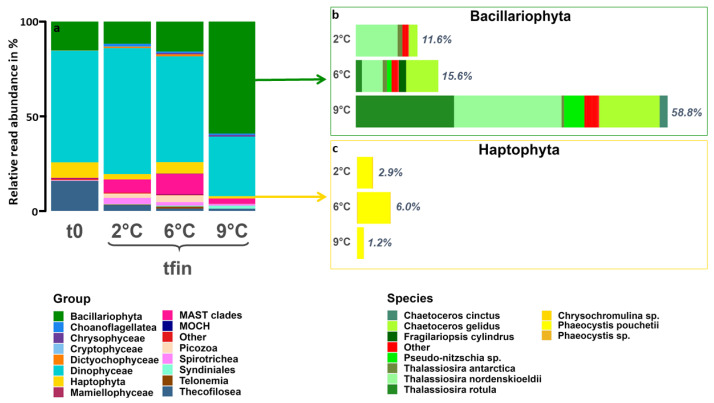
(**a**) ASV-based eukaryotic community composition on class level at the start (t0) and at all treatment temperatures after ten days (tfin). Windows show the relative contribution and species composition of (**b**) Bacillariophyta (green) and (**c**) Haptophyta (yellow) at tfin of all temperature treatments. ASVs with an abundance of fewer than 100 reads among all temperatures were categorized as “other”.

After ten days of incubation, 2 °C and 6 °C showed a more similar composition in comparison to 9 °C (Figure 4a). The 6 °C treatment had slightly higher relative abundances of Choanoflagellatea, Dictyochophyceae, Mammielophyceae, marine Stramenopile (MAST) clades, Picozoa, and Spirotrichea than the 2 °C treatment. In contrast, all these groups were absent or only present in low relative abundances at 9 °C. Furthermore, Bacillariophyta and Syndiniales showed substantially higher relative abundances at 9 °C compared to the two lower temperatures. The dominant group at 2 °C and 6 °C was Dinophyceae, whereas Bacillariophyta dominated at 9 °C. The shifts in relative species abundances of the two main phototrophic classes are shown in Figure 4b,c. The phylum of Bacillariophyta (Figure 4b) comprised the biggest compositional differences among all temperatures. *Thalassiosira nordenskoeldii* was present in all treatments, whereas *Chaetoceros gelidus* had higher relative abundances in the two warming scenarios. *Pseudo-nitzschia* sp., *Chaetoceros cinctus*, and *Thalassiosira rotula* were found in the highest relative abundances at 9 °C but were not detected at 2 °C and were only present in low relative abundances at 6 °C. *Fragilariopsis cylindrus* and *Thalassiosira antarctica* were relatively less abundant or absent at 9 °C. Within the phylum of Haptophyta (Figure 4c), mainly the species *Phaeocystis pouchetii* was present and showed the highest relative abundances at 6 °C, followed by 2 °C and then 9 °C. At 2 °C and 6 °C, *Micromonas polaris* and *commoda* of the class Mammiellophyceae were still present in low relative abundances, whereas they were absent at 9 °C (Figures S5 and S6).

### 3.4. Community Composition—Bacteria

**Figure 5:**
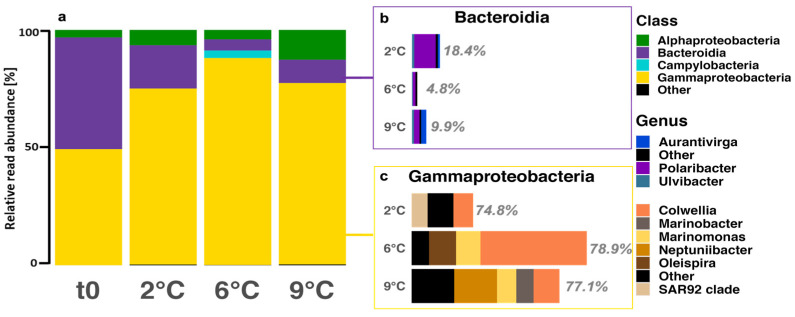
(**a**) ASV-based bacterial community composition on class level at the start (t0) and at all treatment temperatures after ten days (tfin). Windows show the relative contribution and genus composition of (**b**) *Bacteroidia* (purple) and (**c**) *Gammaproteobacteria* (yellow) at tfin of all temperature treatments. ASVs with an abundance of fewer than 100 reads among all temperatures were categorized as “other”.

After ten days, the α- and β-diversity differed among all temperature treatments. As part of the α-diversity analysis, sample completeness using coverage-based rarefaction and extrapolation sampling curves for species richness was greater than 99.97% with 95% confidence intervals. Further interpreting the diversity showed that bacterial richness significantly increased with temperature (ANOVA, F_(3,8)_ = 23.41, *p* < 0.001), while the evenness was significantly higher for the 2 °C and 9 °C treatments than for 6 °C (Table S4). Analyzing the diversity using distance matrices further confirmed significant differences between the temperature treatments, which roughly explains 64% of the total variation (PCoA using Bray–Curtis, MANOVA, *p* = 0.0003). The abundant classes included *Bacteroidia* and *Gammaproteobacteria* (Figure 5a). *Bacteroidia* abundances ranged from 5% to 47% relative abundance, while *Gammaproteobacteria* ranged from 49% to 88% relative abundance. The abundant taxa within *Bacteroidia* included *Polaribacter* (39% to 77%), *Aurantivirga* (7% to 36%), and *Ulvibacter* (8% to 14%). More abundant members within *Gammaproteobacteria* included *Colweilla* (18% to 61%), *Marinomonas* (~13%), the *SAR92* clade (26%), and *Neptuniibacter* (29%).

## 4. Discussion

### 4.1. Warming Induces an Increase in Photoautotrophic, Intermediate-Sized Organisms

While the C:N ratio appeared to be resistant to warming (Tables S3 and S4, see also [66]), the chl*a*:POC ratio significantly increased at 9 °C, indicating either an upregulation of the cellular chl*a* quota of phytoplankton [67] or a higher biomass of phototrophic compared to heterotrophic organisms. Our trophic group data support the latter, as heterotrophy and mixotrophy seem to have been disadvantageous under warming (Figure 3b and Figure S3). While this is in contrast to assumptions made by the metabolic theory of ecology (MTE; [18]), it is supported by Petchey et al. [68], who found a greater extinction frequency of higher trophic positions under warming. A reason for the deviation of our findings from the MTE could be the timespan of the experiments. On short timescales and under nutrient-replete conditions, such as during blooms and in our experiment, fast growers like diatoms may have an advantage over slower-growing heterotrophic and mixotrophic organisms. Predictions by the MTE may only manifest on longer timescales, over years to decades [22]. Additionally, our study only assessed micrograzing and did not account for mesozooplankton, which was removed by the 150 µm mesh (see methods). Increased grazing pressure under warming, as proposed by other authors [69,70], might be restricted to larger organisms and thus could not be accounted for in our experiment. Overall, the cell size and the thermal niche appeared to have been more fundamental than the trophic level for community reorganization under increasing temperatures.

In terms of cell size, warming to 9 °C resulted in a relative increase in intermediate (2–20 µm) as well as a reduction of large (20–150 µm) and small (0.8–2 µm) eukaryotes (Figure 3a and Figure S3). Other studies have also found intermediate-sized organisms to exhibit higher growth rates compared to smaller and larger ones with increasing temperatures [10]. While this is generally in line with the theory of unimodal size scaling of planktonic growth [13], it contrasts predictions from the allometric theory of cell size decreasing with temperature [71]. The observation of this pattern on geological and biogeographical scales but not in controlled experiments could be due to other correlates, such as nutrients and grazing [17]. Nevertheless, results may differ when additional factors related to Atlantification are considered, such as decreased salinity [72]. Interestingly, the increase in intermediate phytoplankton with temperature did not confirm the predicted and modeled growth of small temperate phytoplankton, such as *Emiliania huxleyi* [73] or *Phaeocystis globosa* [74], as these were not present in the field community that we sampled. However, some studies indicate that future Arctic temperatures may not be warm enough for them to be competitive [75,76].

### 4.2. Tipping Point for Arctic Key Eukaryotes Lies between 6 °C and 9 °C

The eukaryotic species’ evenness was similarly high across treatments; thus, no single species dominated the communities (Figure S2). Eukaryotic species richness, however, was lower at 9 °C compared to the other two temperatures, which indicates that much fewer species were able to cope with the higher temperature (Figure S2). A decrease in Arctic phytoplankton richness under warming has also been projected by Benedetti et al. [77], who found temperature to be the main driver of changes in species diversity. Additionally, the phenotypic diversity was significantly higher at 2 °C (Tables S3 and S4), indicating that at 6 °C, the phenotypic characteristics already adapted to the higher temperatures and became more similar. Overall, the lower phenotypic and taxonomic richness found at 9 °C could make the communities more vulnerable to other drivers, as the standing diversity usually increases the communities’ resilience to environmental change [78].

The eukaryotic community composition exhibited the same pattern as the species richness. It was similar between 2 °C and 6 °C, whereas clear qualitative differences could be observed at 9 °C (Figure 4a). This can be attributed to an almost 4-fold higher relative diatom sequence read abundance. Additionally, cosmopolitan species were relatively more abundant at 9 °C, while relatively fewer organisms were detected that could cope with both Arctic and temperate habitats (Figure 3c). Our results suggest an upper thermal limit between 6 °C and 9 °C, which is further supported by other studies observing the growth rates of many Arctic species to decline above 6 °C [79,80]. If future temperatures in the Arctic Ocean reach 9 °C, it may be too warm for Arctic picoplankton and too cold for temperate picoplankton to thrive [81]. Depending on the nutrient conditions, the Arctic may then become favorable for temperate diatoms with comparably high growth rates at lower temperatures [27].

### 4.3. Species-Specific Responses to Warming

Among diatoms, species such as *T. rotula* and *Pseudo-nitzschia* sp. already increased in relative abundance at 6 °C but only started to dominate the community at 9 °C (Figure 4b). Contrastingly, *T. antarctica* and *F. cylindrus* were more prevalent at the two colder temperatures, which is in accordance with their grouping as either Arctic-temperate or solely Arctic, respectively (Table S2). This is supported by a study on the thermal reaction norms of several marine phytoplankton groups [27], which found temperate diatoms drastically increase their growth rates at 10 °C in comparison to 5 °C. Consistently, a study on polar diatoms found that temperatures above 6 °C tend to be supra-optimal for them [82]. Similar results were found for an Arctic *Chaetoceros* strain [83]. However, the intra-specific variation approaching the thermal limits of diatoms appears to be high [82,84], and therefore polar species might adapt to warming in the longer term. Furthermore, one has to keep in mind that the sampling procedure might have excluded larger phytoplankton such as *Coscinodiscus* spp. or long chains and thereby might have skewed these results.

In the Arctic, the genus *Phaeocystis* is predicted to be a ‘climate change winner’ in regards to both warming and Atlantification [85,86,87,88]. Our results refine this prediction by showing that the degree of warming can be critically important in determining the future role of *Phaeocystis* in the Arctic (Figure 4c). While warming to 6 °C in our experiment and to 4.5 °C during a warm water anomaly in the eastern Fram Strait [85] led to a relative increase in the Arctic species *P. pouchettii*, their abundance decreased when temperatures rose further. Similarly, Wang et al. [75] found the upper thermal limit of *P. pouchettii* to be between 8 °C and 12 °C, while the temperate *P. globosa* did not grow below 12 °C. We conclude that if in situ temperatures in the Arctic were to rise above 8 °C but did not reach 12 °C, neither the Arctic nor the temperate *Phaeocystis* species may play a major role unless evolutionary adaptation takes place.

While the cosmopolitan chlorophyte *Micromonas* spp. [89] was only a minor contributor within our starting community, it can dominate the picophytoplankton fraction during summer [90]. At 9 °C, it was completely diminished (no sequence reads left) after the ten days of incubation (Figure S5). Even though it appears to be growing well at 6 °C [91] and has shown a high adaptation potential to warming [92], its population may crash rapidly if temperatures exceed this upper limit. Studies have shown the importance of this genus to overwintering standing stocks and deep-sea export [93,94,95], indicating consequences for the ecosystem if temperatures rise to 9 °C.

Notably, the observed thermal pattern also held true for other organisms, such as the Dictyochophyta, an unidentified Picozoa, as well as several groups of the MAST clade (see also [96]), which all diminished between 6 °C and 9 °C. While the role and importance of these groups are not yet clear, their thermal limits are congruent with the other species that show an Arctic distribution. On the other hand, some organisms (e.g., Syndiniales and Crysophytes) were absent at 2 °C but relatively increased at 6 °C and even more so at 9 °C, which may be indicative of a lower limit of these potentially temperate organisms. These results point towards some kind of universality of the thermal limits between 6 °C and 9 °C found in our study.

### 4.4. Bacterial Diversity and Composition Response to Warming

A notable outcome of our study is linking the bacterial responses to temperature, which is equally important in controlling Arctic bacteria as organic matter [97,98]. However, we must acknowledge that our study is biased towards particle-associated bacteria (>0.8 µm), excluding many smaller free-living bacteria in our analysis [99]. Despite the bias, the dataset allows us to explore details that are usually unavailable for the class *Gammaproteobacteria* compared to *Alphaproteobacteria* and *Bacteroidia* [100].

The microbial community was dominated by *Gammaproteobacteria*, particularly *Colweilla*, with minor contributions by *Bacteroidia*, particularly *Polaribacter* (Figure 5). Throughout the incubation, we observed an increase in the relative abundance of *Colweilla*, peaking at 6 °C, and a decrease in the relative abundance of *Polaribacter* (Figure 5). *Colweilla* likely thrives on the sea ice and terrestrial organic matter during the 2 °C and 6 °C incubation [101,102]. Similarly, *Polaribacter* also thrives on terrestrial organic matter [101] in addition to degrading polymeric organic compounds from phytoplankton [97]. Although *Colweilla* and *Polaribacter* are probably responsible for most of the polysaccharide-derived carbon utilization during the incubation, *Polaribacter* may prove to be more resilient to ongoing changes in the Arctic Ocean, given their ability to respond to phytoplankton-derived or terrestrial organic matter [101].

The phytoplankton community shifted towards temperate diatoms at 9 °C, which affected the microbial richness and evenness. The enhanced presence of diatoms prompted the re-appearance of *Aurantivirga* (Figure 5b), which has been linked to Arctic phytoplankton blooms as early responders to fresh organic matter input [103,104]. Furthermore, *Marinobacter* increased in relative abundance, which is reported to be associated with eukaryotes [105] and enriched in particles [106].

## 5. Conclusions

This study experimentally investigated the potential effect of different degrees of warming on the composition and characteristics of a microbial community in an increasingly Atlantified Arctic Ocean. We uncovered a clear thermal limit for many Arctic phytoplankton species between 6 °C and 9 °C and a concurrent gradual increase in temperate species. Additionally, the bacterial community also changed in response to warming and will, therefore, likely be altered by Atlantification. Our results highlight the importance of the thermal niche for explaining community reorganization under warming as temperate species increasingly invade the Arctic ecosystem. Predictions made by the metabolic theory of ecology that propose heterotrophy to become more prevalent when temperatures rise could not be supported by our experimental set-up and outcome. An intermediate cell size, however, appears to be of advantage, which supports the theory of unimodal scaling of body size. The communities became less diverse in taxonomic richness as well as phenotypic characteristics, leaving them likely more vulnerable to other abiotic changes. Therefore, future studies need to integrate more and different drivers that correlate with the ongoing changes in temperatures. These should include varying nutrient, salinity, and light conditions to account for a more realistic scenario of a future Arctic ecosystem in which temperate organisms may be restricted by other abiotic factors. Overall, our experimental results imply that the future composition of Arctic microbial communities strongly depends on the intensity of warming in the Arctic Ocean.

## Data Availability

The raw CTD data are available from Hoppmann [108]. All data and code used in this article can be found online on GitHub: https://github.com/AntoniaAhme/PS126CommunityExperiment (accessed on 19 January 2023).

## Supplementary Materials

The supporting information can be downloaded at: https://www.mdpi.com/article/10.3390/genes14030623/s1, Table S1: Sequencing statistics from the DADA2 pipeline for all samples after each filtering step and the ratio of final reads to raw reads. The reads containing meaningful taxa were used for downstream analyses; Table S2: Classification of the ASV-based taxonomic groups into three different size classes, four different trophic modes and three different thermal niches. Groups which could not clearly be classified are noted as “uncategorized”; Table S3: Details of biomass and diversity parameters at tfin for each temperature; Table S4: *p*-values of the pairwise t-tests after bonferroni correction for each temperature pair and biomass or diversity parameter; Table S5: Carbonate chemistry and dissolved nutrients of all three treatments at the end of experimental incubation (*n* = 3); Figure S1: The temperature and salinity profile at the sampling site HG-IV. The 15 m sampling depth is marked by the horizontal line. The three dominant water masses in the region (modified after [107]. are indicated by the shaded areas; Figure S2: Replicate-merged bar graphs of the ASV-based class composition after three days for the two unpooled treatments (Unpooled1 = seawater, Unpooled2: 1:5 diluted seawater) and the resulting pools of each temperature that continued the experimental incubation; Figure S3: Bar plot of the mean relative read abundances for each temperature and (a) size groups, (b) trophic modes and (c) thermal niches; Figure S4: RDA plot of the ASVs of each temperature constrained by the biomass and diversity parameters without D2; Figure S5: Relative contribution and species composition of the class of Mammiellophyceae at all treatment temperatures after ten days.
