# Supplementary material for: Winners and Losers of Atlantification: The Degree of Ocean Warming Affects the Structure of Arctic Microbial Communities
Source: Genes (Basel). 2023 Mar 1;14(3):623. doi: 10.3390/genes14030623 (PMC10048660; doi:10.3390/genes14030623)
Supplement: Supplementary file 1 [file genes-14-00623-s001.zip › genes-2222982-supplementary.pdf]

Antonia Ahme, Anabel Von Jackowski, Rebecca A. McPherson, Klara K. E. Wolf, Mario Hoppmann, Stefan Neuhaus, and Uwe John (2023). *Winners and Losers of Atlantification: The Degree of Ocean Warming Affects the Structure of Experimental Arctic Microbial Communities*.

SUPPLEMENTARY MATERIAL

TABLES

Table S1: Sequencing statistics from the DADA2 pipeline for all samples after each filtering step and the ratio of final reads to raw reads. The reads containing meaningful taxa were used for downstream analyses. 2
Table S2: Classification of the ASV-based taxonomic groups into three different size classes, four different trophic modes and three different thermal niches. Groups which could not clearly be classified are noted as "uncategorized". 2
Table S3: Details of biomass and diversity parameters at tfin for each temperature. 4
Table S4: P-values of the pairwise t-tests after bonferroni correction for each temperature pair and biomass or diversity parameter 4
Table S5: Carbonate chemistry and dissolved nutrients of all three treatments at the end of experimental incubation (n=3). 5

FIGURES

Figure S1: The temperature and salinity profile at the sampling site HG-IV. The 15 m sampling depth is marked by the horizontal line. The three dominant water masses in the region (modified after [107] are indicated by the shaded areas. 6
Figure S2: Replicate-merged bar graphs of the ASV-based class composition after three days for the two unpooled treatments (Unpooled1 = seawater, Unpooled2: 1:5 diluted seawater) and the resulting pools of each temperature that continued the experimental incubation. 6
Figure S3: Bar plot of the mean relative read abundances for each temperature and a) size groups, b) trophic modes and c) thermal niches. 7
Figure S4: RDA plot of the ASVs of each temperature constrained by the biomass and diversity parameters without D2. 7
Figure S5: Relative contribution and species composition of the class of Mammiellophyceae at all treatment temperatures after ten days. 8
Figure S6: ASV-based eukaryotic community composition on species level at the start (t0) and at all treatment temperatures after ten days (tfin). ASVs with an abundance of less than 50 reads among all temperatures were categorized as "other". 9

Antonia Ahme, Anabel Von Jackowski, Rebecca A. McPherson, Klara K. E. Wolf, Mario Hoppmann, Stefan Neuhaus, and Uwe John (2023). *Winners and Losers of Atlantification: The Degree of Ocean Warming Affects the Structure of Experimental Arctic Microbial Communities*.

SUPPLEMENTARY TABLES

Table S1: Sequencing statistics from the DADA2 pipeline for all samples after each filtering step and the ratio of final reads to raw reads. The reads containing meaningful taxa were used for downstream analyses.

Sample	Library	Raw	Pre-filtered	Primer-filtered	Quality-filtered	Denoised	Merged	Chimera-filtered	Dominant ASVs	Meaningful taxa	Ratio final/raw
t-0 A	18S rRNA	105972	102129	101733	77265	77221	74725	74167	73760	73732	0.70
t-0 B	18S rRNA	106366	102537	102119	76975	76908	74855	74373	74088	53001	0.50
t-0 C	18S rRNA	115389	111281	110791	84438	84389	81729	80820	80435	80412	0.70
t-fin 2°C A	18S rRNA	161197	155275	154551	113570	113431	109554	107588	107250	92958	0.58
t-fin 2°C B	18S rRNA	150115	144823	144142	112997	112890	109254	107668	107277	107219	0.71
t-fin 2°C C	18S rRNA	149148	143591	142947	109079	108969	105497	103219	102934	92662	0.62
t-fin 6°C A	18S rRNA	139390	134525	133877	100796	100694	97286	95952	95522	95221	0.68
t-fin 6°C B	18S rRNA	146305	141118	140473	107167	107060	103657	102096	101633	99875	0.68
t-fin 6°C C	18S rRNA	134361	129531	128985	95384	95294	92221	91059	90784	76884	0.57
t-fin 9°C A	18S rRNA	70900	68296	68027	51772	51726	49549	49095	48993	48731	0.69
t-fin 9°C B	18S rRNA	101178	97537	97104	74486	74421	71485	70477	70228	69701	0.69
t-fin 9°C C	18S rRNA	55008	52572	52330	31691	31668	30279	29721	29671	29553	0.54
t-0 A	16S rRNA	75650	74686	70544	65573	65500	65048	64791	64497	NA	0.85
t-0 B	16S rRNA	60056	58741	55408	50859	50768	50376	50213	49899	NA	0.83
t-0 C	16S rRNA	85422	82774	76936	71775	71691	71260	70942	70533	NA	0.83
t-fin 2°C A	16S rRNA	71273	68709	64370	59131	59072	58664	58536	58321	NA	0.82
t-fin 2°C B	16S rRNA	53276	50612	47129	44205	44162	43952	43865	43652	NA	0.82
t-fin 2°C C	16S rRNA	49802	48099	45192	42014	41968	41707	41616	41474	NA	0.83
t-fin 6°C A	16S rRNA	60990	60498	55802	52285	52219	51878	51352	51074	NA	0.84
t-fin 6°C B	16S rRNA	62537	62039	57526	54097	54014	53611	53157	52897	NA	0.85
t-fin 6°C C	16S rRNA	64960	64300	59607	55494	55399	54954	54311	53994	NA	0.83
t-fin 9°C A	16S rRNA	60505	57263	53879	50355	50260	49954	49926	49440	NA	0.82
t-fin 9°C B	16S rRNA	72004	68234	63706	60142	60057	59595	59518	59126	NA	0.82
t-fin 9°C C	16S rRNA	33611	33486	30968	28255	28187	27956	27948	27767	NA	0.83

Table S2: Classification of the ASV-based taxonomic groups into three different size classes, four different trophic modes and three different thermal niches. Groups which could not clearly be classified are noted as "uncategorized".

Taxonomic identity	Size	Trophy	Thermal niche	Taxonomic identity	Size	Trophy	Thermal niche
Acanthoecida	uncategorized	heterotroph	Arctic-temperate	MAST-1B	uncategorized	heterotroph	Arctic-temperate
Attheya longicornis	nano	phototroph	Arctic-temperate	MAST-1C	uncategorized	heterotroph	Arctic
Attheya septentrionalis	nano	phototroph	Arctic	MAST-2D	uncategorized	heterotroph	Arctic
Balechina pachydermata	nano	phototroph	Arctic-temperate	MAST-3D	uncategorized	heterotroph	Arctic-temperate
Biecheleria	nano	phototroph	Arctic-temperate	MAST-3I	uncategorized	heterotroph	Arctic-temperate
CCW10-lineage	uncategorized	heterotroph	Arctic	MAST-3L	uncategorized	heterotroph	Arctic-temperate
Chaetoceros cinctus	nano	phototroph	Arctic	MAST-7A	uncategorized	heterotroph	Arctic

Chaetoceros contortus	nano	phototroph	Arctic-temperate
Chaetoceros danicus	nano	phototroph	Arctic-temperate
Chaetoceros debilis	micro	phototroph	Arctic-temperate
Chaetoceros diadema	micro	phototroph	Arctic-temperate
Chaetoceros gelidus	nano	phototroph	Arctic-temperate
Chaetoceros neogracilis	nano	phototroph	Arctic
Chaetoceros peruvianus	micro	phototroph	Arctic-temperate
Chrysochromulina	nano	phototroph	Arctic
Chrysophyceae Clade-C	uncategorized	phototroph	Arctic-temperate
Chrysophyceae Clade-H	uncategorized	phototroph	Arctic-temperate
Chrysophyceae Clade-I	uncategorized	phototroph	Arctic-temperate
Chrysophyceae	uncategorized	phototroph	Arctic
Chytriodinium roseum	nano	parasitic	cosmopolitan
Cryothecomonas aestivalis	nano	mixotroph	Arctic
Cryothecomonas-lineage	uncategorized	mixotroph	Arctic
Detonula confervacea	nano	phototroph	Arctic
Dictyocha speculum	micro	phototroph	Arctic-temperate
Dino-Group-I-Clade-1	nano	parasitic	Arctic-temperate
Dino-Group-I-Clade-4	nano	parasitic	Arctic-temperate
Dino-Group-I-Clade-5	nano	parasitic	cosmopolitan
Dino-Group-II-Clade-1	nano	parasitic	cosmopolitan
Dino-Group-II-Clade-14	nano	parasitic	cosmopolitan
Dino-Group-II-Clade-23	nano	parasitic	Arctic-temperate
Dino-Group-II-Clade-47	nano	parasitic	Arctic-temperate
Dino-Group-II-Clade-52	nano	parasitic	cosmopolitan
Dino-Group-III	nano	parasitic	cosmopolitan
Dinophyceae	uncategorized	uncategorized	cosmopolitan
Dolichomastigaceae-B	uncategorized	phototroph	Arctic-temperate
Ebria tripartita	micro	heterotroph	Arctic-temperate
Eucampia	micro	phototroph	Arctic
Filosa-Thecofilosea	micro	heterotroph	Arctic
Florenciellales	uncategorized	phototroph	Arctic
Fragilariopsis cylindrus	micro	phototroph	Arctic
Fragilariopsis sublineata	micro	phototroph	Arctic
Gymnodinium dorsalisulcum	micro	uncategorized	cosmopolitan
Gymnodinium	micro	uncategorized	cosmopolitan
Gyrodinium dominans	micro	heterotroph	Arctic-temperate
Gyrodinium fusiforme	nano	heterotroph	cosmopolitan
Gyrodinium helveticum	micro	heterotroph	Arctic-temperate
Gyrodinium	uncategorized	heterotroph	Arctic-temperate
MAST-8A	uncategorized	heterotroph	Arctic-temperate
MAST-8D	uncategorized	heterotroph	Arctic-temperate
Mataza-lineage	uncategorized	heterotroph	Arctic
Micromonas commoda	pico	phototroph	Arctic-temperate
Micromonas polaris	pico	phototroph	Arctic
MOCH-2	uncategorized	uncategorized	cosmopolitan
Navicula	uncategorized	phototroph	Arctic-temperate
Naviculaceae	uncategorized	phototroph	Arctic
Nitzschia	uncategorized	phototroph	Arctic
Novel-clade-2	uncategorized	uncategorized	Arctic
Parmales env 3B	uncategorized	mixotroph	Arctic-temperate
Pedinellales	uncategorized	mixotroph	Arctic-temperate
Pentapharsodinium	uncategorized	uncategorized	Arctic
Pentapharsodinium tyrrhenicum	micro	parasitic	Arctic
Phaeocystis pouchetii	nano	phototroph	Arctic
Phaeocystis	nano	phototroph	cosmopolitan
Picozoa	Pico	heterotroph	Arctic-temperate
Plagioselmis prolonga	nano	phototroph	cosmopolitan
Pleurostomatida	uncategorized	heterotroph	Arctic
Polarella glacialis	nano	phototroph	Arctic
Porosira glacialis	micro	phototroph	Arctic
Prorocentrum cordatum	nano	mixotroph	Arctic-temperate
Prorocentrum	uncategorized	uncategorized	cosmopolitan
Protaspa-lineage	uncategorized	heterotroph	Arctic
Prymnesiophyceae Clade E	uncategorized	uncategorized	Arctic
Pseudo-nitzschia delicatissima	micro	phototroph	Arctic-temperate
Pseudo-nitzschia multiseries	micro	phototroph	cosmopolitan
Pseudo-nitzschia seriata	micro	phototroph	Arctic-temperate
Pseudo-nitzschia	micro	phototroph	cosmopolitan
Pterosperma cristatum	nano	phototroph	uncategorized
Pyramimonadales	uncategorized	phototroph	Arctic-temperate
Rhizosolenia imbricata shrubsolei	micro	phototroph	Arctic-temperate
Scrippsiella acuminata	micro	mixotroph	Arctic-temperate
Skeletonema marinoi	nano	phototroph	Arctic-temperate
Stephanoecidae Group D	uncategorized	heterotroph	Arctic-temperate
Stephanoecidae Group H	uncategorized	heterotroph	Arctic-temperate
Stramenopiles	uncategorized	heterotroph	Arctic-temperate
Strobilidiidae A	uncategorized	heterotroph	Arctic
Strombidiida B	uncategorized	heterotroph	Arctic-temperate
Strombidiida F	uncategorized	heterotroph	Arctic-temperate

Gyrodinium spirale	micro	heterotroph	Arctic-temperate
Haptophyta Clade HAP5	uncategorized	phototroph	cosmopolitan
heterotrophcapsa nei/rotundata	nano	mixotroph	cosmopolitan
heterotrophcapsa pygmaea	nano	uncategorized	Arctic-temperate
Hypotrichia	uncategorized	heterotroph	Arctic
Islandinium minutum	micro	heterotroph	Arctic
Islandinium tricingulatum	micro	heterotroph	Arctic
Karlodinium veneficum	nano	mixotroph	Arctic-temperate
Laboea strobila	micro	mixotroph	Arctic-temperate
Labyrinthulomycetes LAB14	uncategorized	uncategorized	Arctic
Leegaardiella	micro	heterotroph	Arctic-temperate
Mantoniella squamata	nano	mixotroph	Arctic-temperate
MAST-12A	uncategorized	heterotroph	Arctic-temperate
MAST-1A	uncategorized	heterotroph	Arctic-temperate
Strombidiidae H	uncategorized	heterotroph	Arctic
Strombidiidae M	uncategorized	heterotroph	Arctic-temperate
Strombidinopsis	uncategorized	heterotroph	Arctic-temperate
Strombidium capitatum	micro	heterotroph	Arctic
TAGIRI1-lineage	uncategorized	heterotroph	Arctic
Teleaula gracilis	nano	mixotroph	cosmopolitan
Telonemia-Group-1	uncategorized	uncategorized	Arctic-temperate
Thalassiosira antarctica	nano	phototroph	Arctic
Thalassiosira concaviuscula	uncategorized	phototroph	Arctic-temperate
Thalassiosira hispida	uncategorized	phototroph	Arctic
Thalassiosira nordenskioeldii	nano	phototroph	Arctic-temperate
Thalassiosira rotula	micro	phototroph	Arctic-temperate
Ventricleftida	uncategorized	heterotroph	Arctic
Not identified	uncategorized	uncategorized	uncategorized

Table S3: Details of biomass and diversity parameters at tfin for each temperature.

Temp. [°C]	Chl*a* [µg/L]	POC [µg/L]	PON [µg/L]	Chl*a*:POC [g:g]	C:N [mol:mol]	D2	RichnessE	EvennessE	RichnessP	EvennessP
**2**	1.9 ± 0.6	74 ± 7.8	15.3 ± 2	0.03 ± 0.01	5.6 ± 0.7	8815 ± 222	211 ± 3	0.59 ± 0.01	180 ± 8	0.45 ± 0.04
**6**	2.5 ± 0.6	153.6 ± 84	30.8 ± 15.4	0.02 ± 0.01	5.8 ± 0.5	6641 ± 788	218 ± 2	0.63 ± 0.02	201 ± 2	0.55 ± 0.01
**9**	24.4 ± 24.7	312.4 ± 274.8	56.9 ± 52.5	0.09 ± 0.05	6.4 ± 0.2	6018 ± 529	124 ± 21	0.61 ± 0.04	179 ± 19	0.54 ± 0.04

Table S4: P-values of the pairwise t-tests after bonferroni correction for each temperature pair and biomass or diversity parameter

Pairs	Chl*a*:POC	C:N	D2	RichnessE	EvennessE	RichnessP	EvennessP
**2°C-6°C**	0.751	1	0.072	1	0.218	0.253	0.032 *
**2°C-9°C**	0.048 *	0.433	0.008 **	0.001 **	0.88	1	0.065
**6°C-9°C**	0.011 *	0.749	0.437	0.001 **	1	0.213	1

Antonia Ahme, Anabel Von Jackowski, Rebecca A. McPherson, Klara K. E. Wolf, Mario Hoppmann, Stefan Neuhaus, and Uwe John (2023). *Winners and Losers of Atlantification: The Degree of Ocean Warming Affects the Structure of Experimental Arctic Microbial Communities*.

Table S5: Carbonate chemistry and dissolved nutrients of all three treatments at the end of experimental incubation (n=3).

**Temp. [°C]**	**pH**	**TA [µmol/kg]**	**TA drift to t-0 [%]**	**pCO2 [µatm]**	**HCO3 [µmol/kgSW]**	**CO3 [µmol/kgSW]**	**CO2 [µmol/kgSW]**	**DIC [µmol/kgSW]**	**PO4 [µmol/L)**	**SiO4 [µmol/L]**	**NO2 [µmol/L]**	**NO3 [µmol/L]**	**NH4 [µmol/L]**
**2**	8.09 ± 0.0	2260 ± 6	0.7	373 ± 1	2004 ± 6	98.1 ± 0.3	21.8 ± 21.8	2124 ± 6	4.9 ± 0.1	32.1 ± 0.6	0.1 ± 0	62.8 ± 2.2	0.7 ± 0.1
**6**	8.04 ± 0.0	2260 ± 3	0.1	512 ± 55	2028 ± 17	89.3 ± 7.7	23.7 ± 25.8	2144 ± 12	4.9 ± 0.1	32.1 ± 0.3	0.1 ± 0	63.0 ± 1.7	0.7 ± 0.3
**9**	8.15 ± 0.1	2260 ± 10	-0.1	440 ± 107	1966 ± 53	113.0 ± 24.1	24.4 ± 20.0	2101 ± 34	4.7 ± 0.1	25.8 ± 4.1	0.1 ± 0	57.5 ± 5.4	0.2 ± 0

Antonia Ahme, Anabel Von Jackowski, Rebecca A. McPherson, Klara K. E. Wolf, Mario Hoppmann, Stefan Neuhaus, and Uwe John (2023). *Winners and Losers of Atlantification: The Degree of Ocean Warming Affects the Structure of Experimental Arctic Microbial Communities*.

SUPPLEMENTARY FIGURES

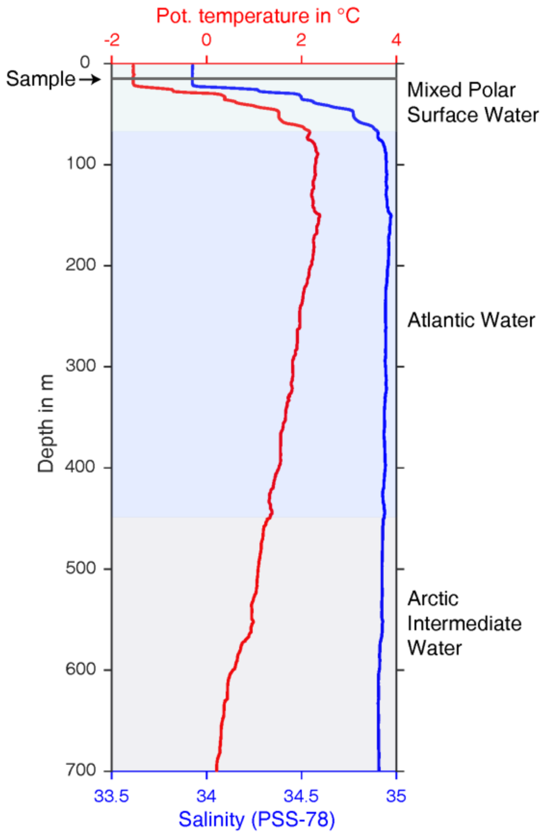

Figure S1: The temperature and salinity profile at the sampling site HG-IV. The 15 m sampling depth is marked by the horizontal line. The three dominant water masses in the region (modified after [107] are indicated by the shaded areas.

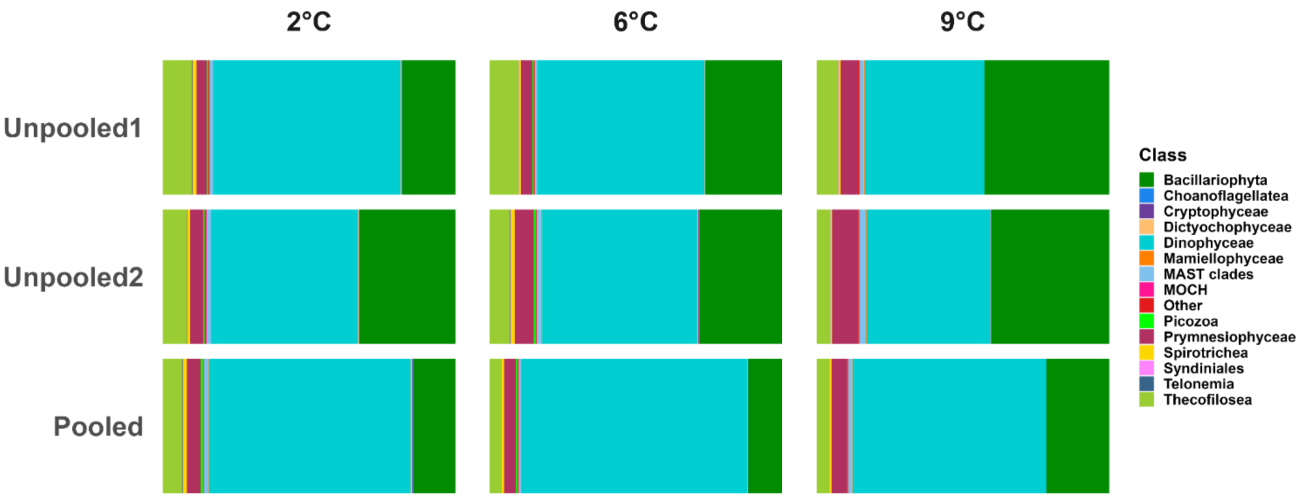

Figure S2: Replicate-merged bar graphs of the ASV-based class composition after three days for the two unpooled treatments (Unpooled1 = seawater, Unpooled2: 1:5 diluted seawater) and the resulting pools of each temperature that continued the experimental incubation.

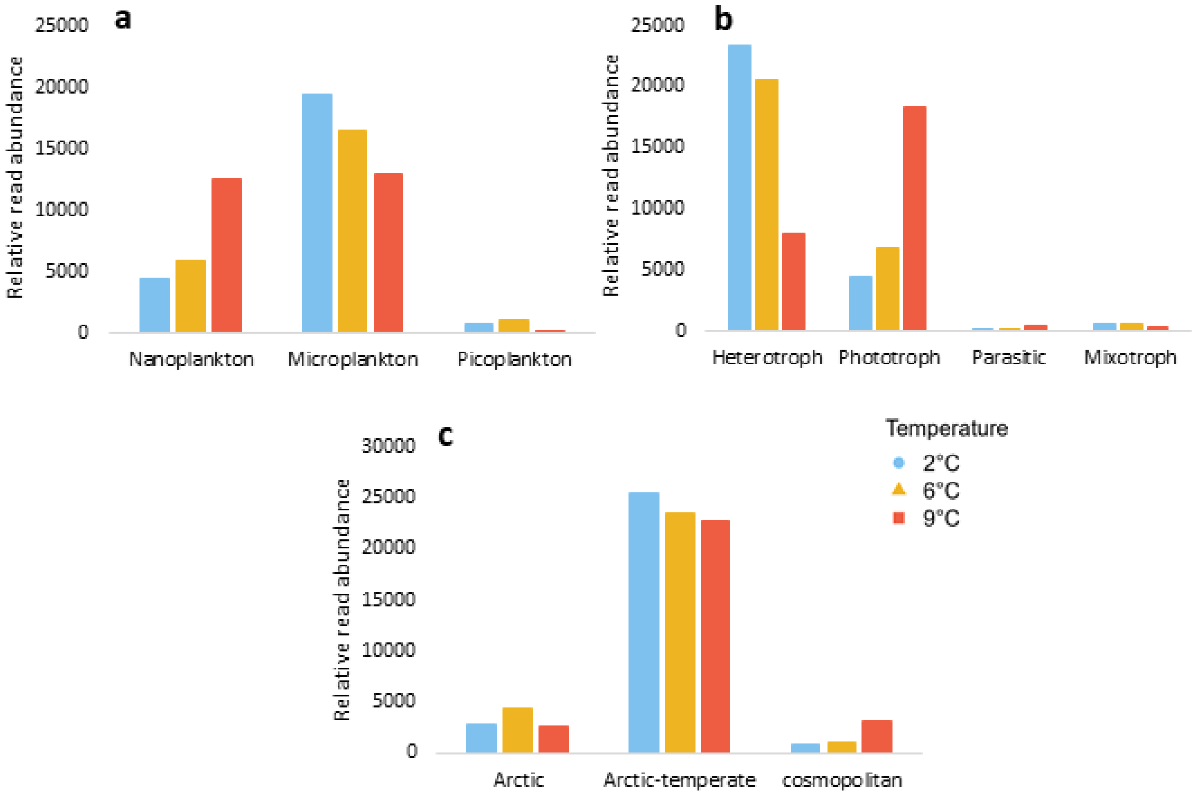

Figure S3: Bar plot of the mean relative read abundances for each temperature and a) size groups, b) trophic modes and c) thermal niches.

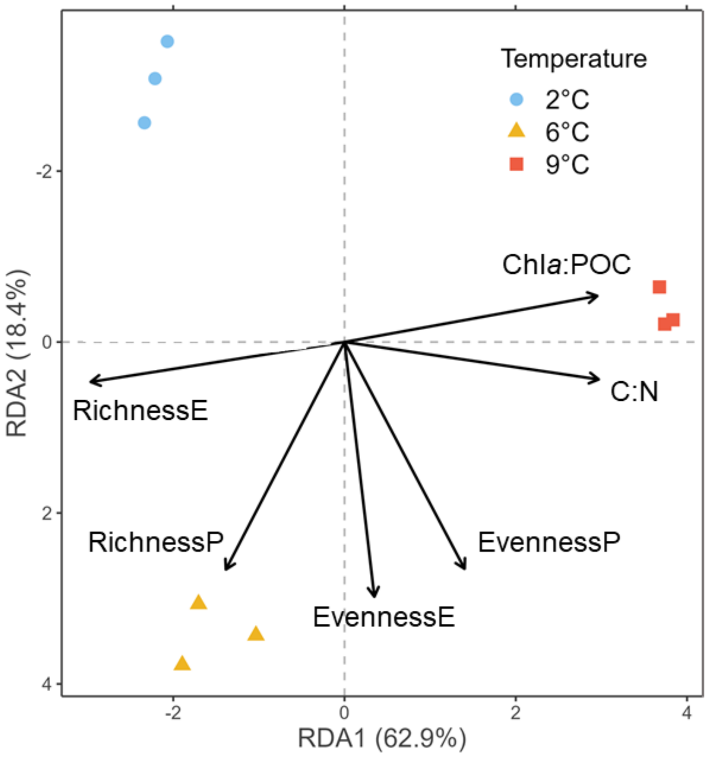

Figure S4: RDA plot of the ASVs of each temperature constrained by the biomass and diversity parameters without D2.

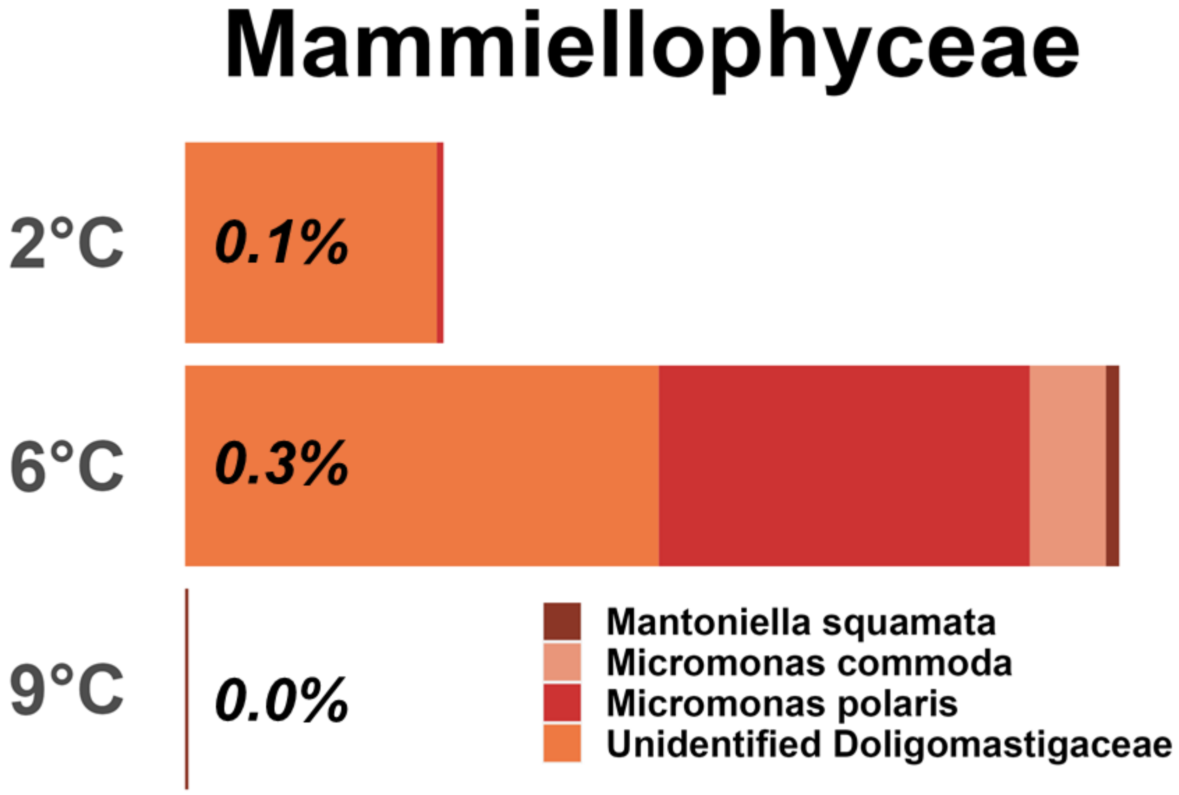

Figure S5: Relative contribution and species composition of the class of Mammiellophyceae at all treatment temperatures after ten days.

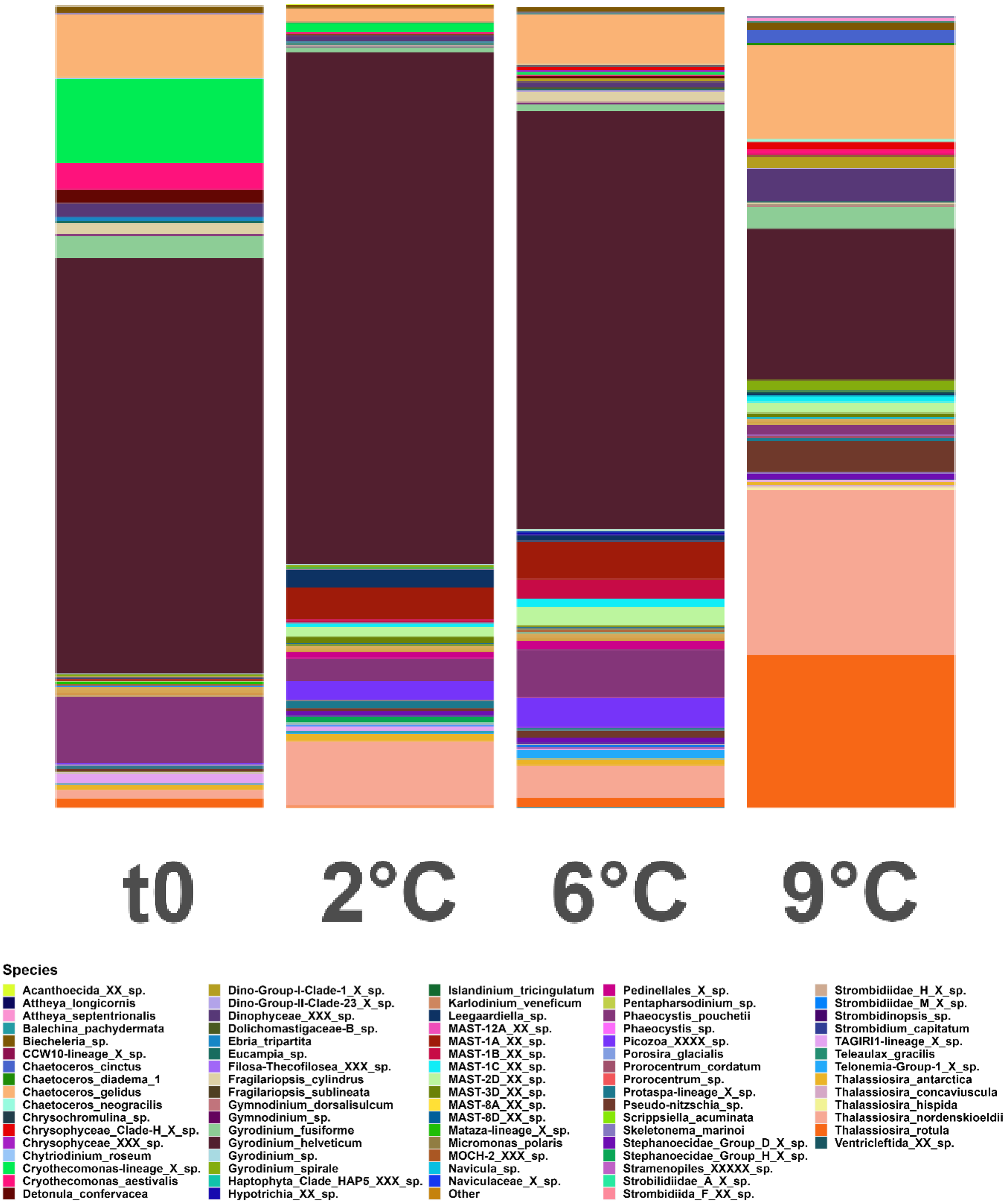

Figure S6: ASV-based eukaryotic community composition on species level at the start (t0) and at all treatment temperatures after ten days (tfin). ASVs with an abundance of less than 50 reads among all temperatures were categorized as "other".
